# Why help others? Insights from rodent to human early childhood research

**DOI:** 10.3389/fnbeh.2023.1058352

**Published:** 2023-03-21

**Authors:** Ya-Qin Chen, Shu Han, Bin Yin

**Affiliations:** ^1^Laboratory of Learning and Behavioral Sciences, School of Psychology, Fujian Normal University, Fuzhou, Fujian, China; ^2^Department of Applied Psychology, School of Psychology, Fujian Normal University, Fuzhou, Fujian, China

**Keywords:** helping behavior, motivation, comparative cognition, rodents, primates, human early childhoods

## Abstract

Helping behavior are actions aiming at assisting another individual in need or to relieve their distress. The occurrence of this behavior not only depends on automated physiological mechanisms, such as imitation or emotional contagion, that is, the individual’s emotion and physiological state matching with others, but also needs motivation to sustain. From a comparative and developmental perspective, we discover that the motivation for helping behavior has a deep foundation both phylogenetically and ontogenetically. For example, empathic concern for others, relieving personal distress and the desire for social contact are universal motivations across rodents, non-human primates and human early childhoods. Therefore, a circle-layered model integrating evidences for motivation for helping behavior from rodent to human early childhood research is proposed: the inner circle contains the emotional-behavioral system and the outer circle contains the affective-cognitive system. The application of this model has significance for both behavioral neuroscience research and cultivating prosocial behavior in human society.

## 1. Phenomenon: Helping behavior is universal across species

The debate on whether human nature is inherently good has been discussed for thousands of years. In the first and second years of life, human infants will try to understand the distress of others, and show their concern for the distress of others by embracing and patting (Zahn-Waxler et al., 1992), which means that even infants who learn to speak in babbling can also help others. For a long time, scholars have been thinking about whether these behaviors are inherent in human beings, and also triggered the thinking about “whether the helping behavior is unique to human beings.” Of course, the answer is no. Studies have found that helping behavior exists not only in primates, carnivores, rodents, and other mammals, but also in birds, fish, and other non-mammal vertebrates. Lome, a chimpanzee, refused to enjoy the food alone and shared it with his peers in a friendly way (Schmelz et al., 2017). Following a conflict among chimpanzees, bystanders often engage in a post-conflict affiliative behavior, referred to as “consolation,” wherein they provide comfort to the recipient of aggression by grooming their hair, in order to alleviate their distress (Fraser and Aureli, 2008). Bonobos can comfort their defeated companions through physical contact (Clay and de Waal, 2013a,b). Domestic dogs can also cooperate in problem solving situations to obtain food (Bräuer et al., 2013). Wolves will breed cooperatively to help protect unrelated pups, which is affected by individual and population characteristics (Ausband et al., 2016). Free rats will rescue their companions trapped in a narrow space (Bartal et al., 2011, 2014, 2016; Havlik et al., 2020) or falling into the water (Sato et al., 2015; Kandis et al., 2018; Cox and Reichel, 2020). In addition, when there is a predator risk, purple crowned fairy wrens decide whether to help and cooperate to defend predators based on social background (interests and kinship) (Kern, 2021; Teunissen et al., 2021). The cleaner fish (*Labroides dimidiatus*) will help their companions to remove parasites from their bodies (Bshary and Grutter, 2006). In cooperatively-breeding cichlid snapper, unrelated individuals will need to bring more help to new breeders in order to be protected by the population or allowed to enter the breeding territory (Balshine-Earn et al., 1998; Stiver et al., 2005). These findings suggest that helping behavior may be universal across species and may be older than the emergence of human beings in phylogeny.

## 2. Definition and potential mechanisms: An overview

Helping behavior is a crucial form of prosocial behavior that involves actions intended to assist another person with a problem or to alleviate their distress (Stukas and Clary, 2012). Such behavior is inherently others-oriented and beneficial to the recipient (Mason, 2021). Based on the nature of the assistance provided, helping behavior can be classified into instrumental helping and emotional helping (Bamberger et al., 2017). Instrumental helping is directed toward providing specific, tangible, or goal-oriented assistance, and is aimed at helping the recipient complete a task or achieve a goal. On the other hand, emotional helping involves the sharing of feelings, demonstrating sympathy, caring, understanding, and friendship, among other forms of emotional support. Thus, caring, comforting, and encouraging others can also be considered as forms of helping behavior. Successful helping behavior includes the interaction and influence between helpers and help seekers (Fischer et al., 2006; Rand and Epstein, 2014). On one hand, helpers need to pay attention to the needs of help seekers before providing help; on the other hand, the urgency of the need of the help seekers will also affect the helping behavior. In emergency situations, people often provide help without thinking, while the presence of others may inhibit the occurrence of helping behavior [the bystander effect, see (McFarland and Majolo, 2012; Plötner et al., 2015; Havlik et al., 2020)]. In addition, in the process of helping, the ability to perceive the signal of others in need of help may be critical for the motivation for helping behavior (Decety et al., 2016). The distress signal is often a strong call for action, and the tendency to perceive the distress of others is deeply rooted in evolution - many species can show sensitivity to the emotional state of each other’s distress and make appropriate responses accordingly. In rodents, hearing the 22 kHz alarm call of others can induce the freezing of observer rats (Parsana et al., 2012). Among bonobos, after some social conflict, bystanders will approach the defeated party, hug the screaming conspecific and groom for them, exhibiting comforting behavior (Clay and de Waal, 2013a,b). Similarly, newborn infants often respond to the cries of other infants with their own cries (Sagi and Hoffman, 1994). This phenomenon demonstrates the complex foundation of empathy in many mammals, from rodents to humans—the perception-action mechanism (de Waal and Preston, 2017).

Preston and Hofelich (2012) believe that empathy should be understood from a broader perspective. Emotional contagion, sympathy, cognitive empathy, and helping are phenomena related to empathy, which all depend on the process of perception-action mechanism (PAM). In addition, there are phenomena such as imitation, yawning, automation, etc., which are based on the same mechanism and are coherent into a whole under the PAM model. In particular, the model emphasizes that the participation in and the perception of the object state will automatically activate the representation of the object’s state and situation in the subject, so as to arouse the matched emotional state and produce appropriate behavioral response (Preston and de Waal, 2002). Take human beings as an example, that is, when the emotional response to the distress of others stimulates the motivation to act for the benefit of others, individuals tend to initiate prosocial behavior (Meyza et al., 2017).

In order to investigate the evolutionary roots of helping behavior, this paper provides a review of research on helping behavior in rodents, non-human primates, and human infants and toddlers. These three species are typical models for studying the evolutionary roots of helping behavior. Human infants, who exhibit a low degree of socialization and typically act spontaneously, non-human primates, who are close relatives of humans and exhibit similarities in behavior and cognition, and rodents, one of the oldest mammalian groups, which are often used to study the neural mechanisms of pro-social behavior, are all devoid of strict and clear cultural rules or codes of conduct, thereby excluding the influence of cultural factors on helping behavior (Melis, 2018). Furthermore, these three species have been shown to respond to each other’s distress, indicating the potential existence of shared psychological mechanisms for helping. Comparative research on these three species will promote a better understanding of the complex and dynamic developmental processes (Lickliter and Bahrick, 2007) and clarify the roots of helping behavior. By summarizing the literature on rodent helping behavior, we found various explanations and debates surrounding the motivation for rodent helping behavior, such as altruism to empathize with others, egoism to alleviate their own distress, and the pursuit of social contact (Bartal et al., 2011, 2014; Schwartz et al., 2017; Carvalheiro et al., 2019; Blystad, 2021). This review aims to summarize and discuss the three types of helping behavior motivation from a cross-species perspective. Based on these evidences, a circle-layered model of helping-behavior motivation will be proposed, which incorporates both proximal and ultimate causes. The model will provide a framework to explain and predict the motivation of helping behavior from an evolutionary and developmental perspective.

## 3. Why help others: Concern for others

The unsolicited and active helping behavior often depends on the spontaneity of the helper and the real concern for the welfare of others (Chou and Stauffer, 2016). When someone really cares about the welfare of others, helping is for the benefit of others rather than oneself. Therefore, the motivation of helping is altruistic, just as Webster (1944) defined altruism as “respect and dedication to the interests of others.” In addition, caring for others, altruism and empathy are closely related to each other, and the process related to empathy induces pro-social behaviors such as helping others and caring for others (Decety and Cowell, 2014).

### 3.1. Evidences from studies on rodents

**FIGURE 1:**
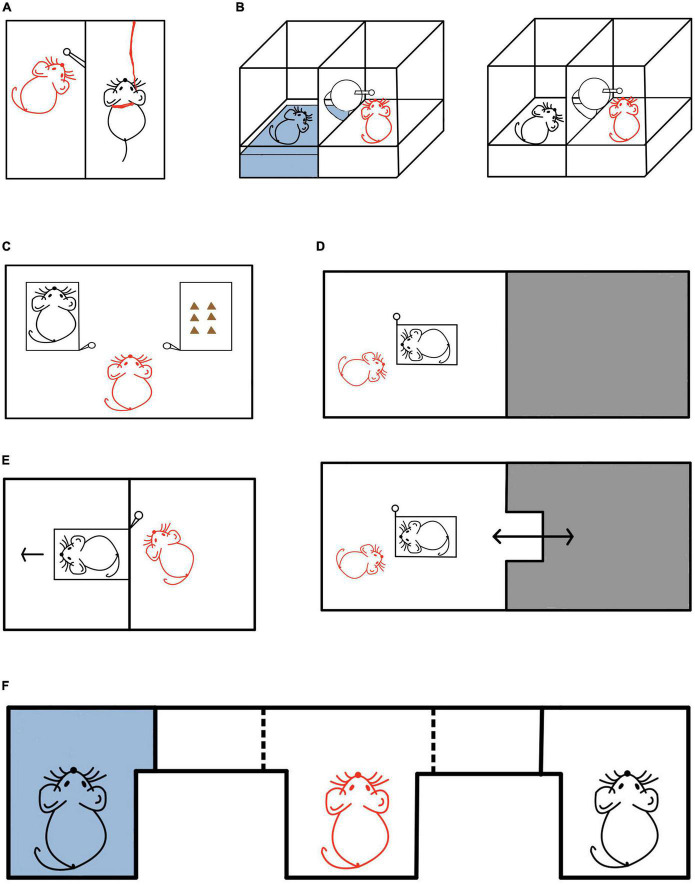
Helping behavior experiments in rodents. **(A)** In this experiment, a black rat was in distress and a red rat was the free rat (helper). The free rat had the ability to press the lever bar to rescue the distressed rat, who was suspended in the air by a harness (Rice and Gainer, 1962). **(B)** In this experiment, a black rat was either in distress or not, and a red rat was the free rat (helper). When the distressed rat was trapped in the water (left panel), the free rat could learn to rescue the trapped distressed rat by pulling the lever. As sessions progressed, the latency to help decreased. In contrast, the free rat did not pull the lever when the rat was not in distress in the ground area (right panel) (Sato et al., 2015). **(C)** In this experiment, a black rat was trapped in the left restrainer and in need of help, with some chocolate in the right restrainer. The red rat was the free rat (helper). When faced with the choice of rescuing the conspecific and enjoying the delicious food, the free rat chose to first rescue the trapped conspecific and share the food with it (Bartal et al., 2011). **(D)** In this experiment, a black rat was trapped in the restrainer and in need of help, and the red rat was the free rat (helper). The gray area was the darkroom that allowed the free rat to escape from the helping situation. In the upper panel, the darkroom door is closed, and the free rat cannot enter. But in the lower panel, the darkroom door is open, and the free rat can enter. Compared with rats in the non-escapable group (upper panel), rats in the escapable group (lower panel) had a longer delay in opening the door to help rescue the trapped rat (Carvalheiro et al., 2019). **(E)** In this experiment, a black rat was trapped in the restrainer and in need of help, and the red rat was the free rat (helper). Even when unable to make social contact after helping, the free rat still chose to open the door for the trapped rat in the restrainer (Bartal et al., 2011). **(F)** In this experiment, the black rats in need of help were placed at both ends of the E maze (left end: the rat was trapped in the wet goal box; right end: the rat was trapped in the dry goal box). The red rat in the middle was the free rat (helper) and could choose freely. It was found that the free rat preferred to interact with the rat in the wet goal box (Schwartz et al., 2017).

Whether the motivation of helping others in rodents is based on caring for the conspecific (empathy) has attracted the attention of many contemporary scholars (Bartal et al., 2011, 2014; Sato et al., 2015; Cox and Reichel, 2020). As early as the 1960s, Rice and Gainer (1962) showed that rats in free state (hereinafter referred to as “free rats” or “observer rats”) would help trapped rat who was shocked or suspended by a harness (hereinafter referred to as “distressed rats “) in another chamber by pressing the bar (Figure 1A). Studies in the past 10 years have shown that free rats will constantly try to rescue cage-mates trapped in a narrow space (restrainers) (Bartal et al., 2011, 2014, 2016, 2021), and they can distinguish the targets between their conspecific and objects. Under the conditions of toy rat and empty restrainer, the helping behavior by opening the door becomes less (Bartal et al., 2011), which Bartal et al. (2011, 2014) explained as the empathetic ability of rodents to the conspecific in distress. Similarly, in the experiment of Sato et al. (2015) (Figure 1B), in order to further clarify that the helping behavior of rats is caused by the distress state of their cage-mates, the rat was placed either in a pool area (water can soak the rat, causing serious disgust or distress) or in a ground area without water. It was found that when the rat was trapped in the pool area, the free rat would learn to help by opening the door and the latency to help became shorter and shorter as sessions went. However, when the rat was in the ground area with no distress, the free rat would not open the door to help, indicating that perceived distress of their peers is a necessary condition for helping to occur. Rats with soaking experience learned to help faster than those without soaking experience, so it is believed that the helping behavior of rats was due to empathy with other individual with similar experiences. However, Cox and Reichel (2020) suspected that in previous studies, trapped rats could be socially contacted by helpers in the same environment after being rescued, thus the study could not distinguish whether helping behavior was driven by empathy or the desire for social contact. In order to exclude the possibility of social interaction, on the basis of Sato et al. (2015), Cox and Reichel (2020) added a dry compartment so that the rescued rats could only exit to the dry compartment but could not make contacts with the helper. The results demonstrated that the free rat would still pull the chain to rescue the distressed conspecific, and the helping behavior continued over time, with the latency to help less and less. Importantly, the latency to help only got shorter when the conspecific is trapped but got longer when the wet compartment was empty or a fake rat (or toy rat) was inside instead of the conspecific. These data were believed to support the notion of the impact of “direct” empathic concerns for their conspecific, though strongly modulated by efforts as increasing the difficulty of helping significantly decreased such behavior. The experiment supports the PAM model of empathy, i.e., the existence of conspecific in distress can promote state matching and thus trigger helping behavior.

Similar social experience can promote the emotional empathy response to the distress of the conspecific (Eklund et al., 2009; Atsak et al., 2011; Iwasaki et al., 2015). Sanders et al. (2013) used the fear conditional paradigm and found that mice who had been shocked before were more frightened when they saw their peers being shocked. The experiment of Sato et al. (2015) also supported this result. during the role reversal stage, the reverse observer (formerly trapped rat) had significantly shorter latency to help. These data suggest that similar stressful experience in the past can promote state matching and facilitate helping behavior.

In order to further quantify the value of empathetic helping, Bartal et al. (2011) and Sato et al. (2015) set different amount of food rewards as opportunity costs for helping the conspecific. In Bartal et al.’s (2011) experiment (Figure 1C), when the rats were forced to choose between helping the trapped cagemate by opening the door of one restrainer and getting the food reward by opening the door of another restrainer, most of the rats would choose to help the cagemate before getting the food rewards, indicating that the value of helping the cagemate was greater than the value of the food rewards. The experiment of Sato et al. (2015) also supported this result. Similarly, Kitano et al. (2022) has recently shown that prairie voles would help to rescue their conspecifics from soaking in water. When their conspecifics were not soaking in water, prairie voles’ door opening behavior were inhibited. In the latest study, Breton et al. (2022) proved that the young rats would show helping behavior to both members of their ingroup and members of the outgroup. By observing the neural activity of the whole brain of the rats, they found that when the target in need of help is members of the ingroup, the neural areas related to empathy are more active.

In conclusion, in the above studies, scholars believe that the helping behavior of rodents is based on the empathic concern for the conspecific’s state of distress. Free rats can sense the distress of the conspecific through the emotional contagion process and help to alleviate the distress of the conspecific (Bartal et al., 2011, 2014; Sato et al., 2015; Cox and Reichel, 2020).

### 3.2. Evidences from studies on non-human primates

**FIGURE 2:**
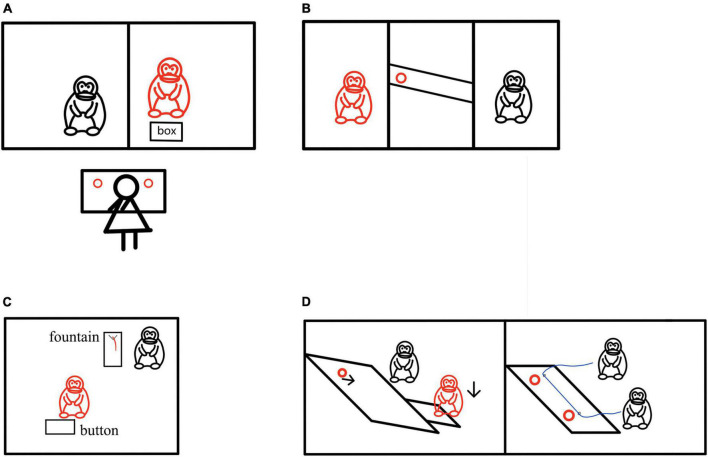
Helping behavior experiments in non-human primates. **(A)** The red chimpanzee acted as the subject, the black chimpanzee as the companion, and figure in black was the experimenter. The red circle represented the food reward. The pro-social selection paradigm was employed where the subject was given a box of tokens that could be exchanged for food. Tokens included pro-social and pro-self tokens, with their meanings taught beforehand. Pro-social tokens resulted in rewards for both the subject and their companion, whereas pro-self tokens only yielded a reward for the subject. Results showed that chimpanzees predominantly chose pro-social tokens (Horner et al., 2011). **(B)** The red chimpanzee acted as the subject, and the black chimpanzee was either a friend or neutral peer. The red circle represented the food reward. The subjects were familiar with using tools to deliver food beforehand. During the test, they could choose to pull the rope to deliver food to either their friend or the neutral peer. Results showed that chimpanzees preferred to help their friends obtain food (Engelmann et al., 2019). **(C)** The red chimpanzee acted as the subject, and the black chimpanzee was the recipient. When the subject pressed the button, juice would flow out of a distant fountain, but only the recipient could drink it. Results showed that the majority of chimpanzees would continuously press the button (van Leeuwen et al., 2021). **(D)** The red marmoset acted as the subject, and the black marmoset was the recipient. The red circles represented the food reward. The altruistic paradigm on the left showed that when the subject stepped on the platform, only the black marmoset next to it received food. The cooperative paradigm on the right showed that when the red and black marmosets pulled the rope simultaneously, they could obtain food together (Martin et al., 2021).

In the study of helping behavior of non-human primates, evidence shows that the motivation of helping may also be altruistic (Yamamoto et al., 2009; Baden et al., 2013). When the human experimenter pretends to be unable to reach some items and needs help, the chimpanzee can even help the human experimenter to get the items before the human experimenter makes a request, even if there is no benefit in doing so (Warneken and Tomasello, 2006). In a pro-social choice paradigm, chimpanzees could choose pro-self tokens from the box to get food for themselves, or choose pro-social tokens to get food for themselves and their peers (Figure 2A). Most of the subjects chose pro-social tokens, indicating that chimpanzees would spontaneously help without the request of their peers (de Waal, 2007; Horner et al., 2011). Engelmann et al. (2019) explored whether chimpanzees are more motivated to help their friends. In the experiment, chimpanzees were first trained to use tools to deliver food. In the test stage, they could pull the tether to deliver food either to their friends or to neutral peers, and provide help to each other (Figure 2B). The results showed that chimpanzees prefer to help friends. In recent studies, in order to explore whether capuchins also have altruistic behavior, researchers first trained capuchins to learn to obtain keys to obtain food, and then conducted a sharing test and a helping test on them to study the altruistic behavior of capuchins. In the sharing test, capuchins can open the door to share food with other capuchins, while in the helping test, they can help other capuchins to obtain the key to obtain food. The research results showed that although capuchin monkeys are unwilling to share food, they will help another capuchin to get food (Bucher et al., 2021). van Leeuwen et al. (2021) observed the prosocial behavior of chimpanzees. In the study, chimpanzees could press the button to make the distant fountain flow juice, thus benefiting the conspecific near the fountain (Figure 2C). However, pressing the button was not beneficial for the subjects themselves. The results demonstrated that most chimpanzees participating in the experiment show continuous and even increasing prosocial behavior.

In addition, Martin et al. (2021) explored whether the prosocial motivation in marmosets could increase the possibility of cooperation through an altruistic paradigm and a cooperative paradigm (Figure 2D). In an altruistic paradigm, when the red marmosets stepped on the platform, the black marmosets could get food, while the red marmosets could not; while in the cooperative paradigm, when red marmosets and black marmosets pulled the string at the same time, they could get food. The results showed that the prosocial dyads were much more likely to achieve successful cooperation than the non-prosocial dyads. Recent studies have found that female wild bonobos would adopt and take care of cubs outside the group (Tokuyama et al., 2021). They would even demonstrate maternal love behavior to these outgroup cubs, including carrying on their backs, grooming, breastfeeding, nesting, lasting more than a year. Wild chimpanzees also adopt orphans from relatives (Hobaiter et al., 2014; Reddy and Mitani, 2019), and even infants from other groups (Casar and Young, 2008).

In conclusion, from the above research, non-human primates may also have the motivation of caring for their conspecific and demonstrate altruistic helping behavior.

### 3.3. Evidences from studies on human early childhoods

**FIGURE 3:**
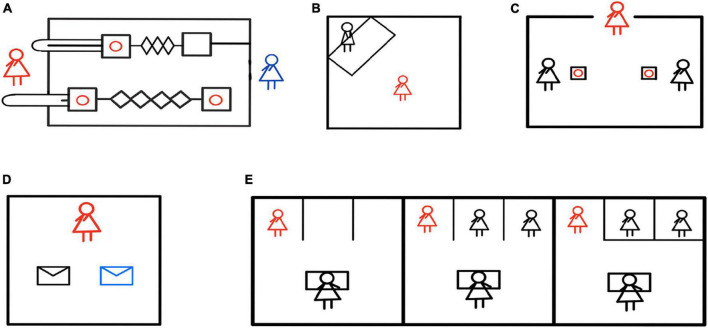
Helping behavior experiments in human early childhoods. **(A)** The red figure represented the toddler actor, and the blue figure represented the toddler receiver. The red circle was the reward. The actor could choose to pull the lower pro-social pull rod, which would benefit both themselves and the receiver, or the upper pro-self pull rod, which would only benefit themselves. The experiment found that 2.5-year-old toddlers pulled more pro-social levers, indicating a preference for pro-social behaviors (Sebastián-Enesco et al., 2013). **(B)** The red figure represented the infant actor, and the black figure represented the experimenter. The experiment observed the infants’ instrumental helping behavior when the experimenter pretended that something had fallen and required assistance. The study found that additional rewards decreased the infant’s helping behavior (Warneken and Tomasello, 2008). **(C)** The red figure represented the actor, and the black figure represented the receiver. The experiment investigated whether children would help their friends or peers when given a choice to assist only one. The results revealed that young children helped their friends more but also helped their non-friend peers (Engelmann et al., 2019). **(D)** The red figure represented the actor, who had ten stickers that could be kept or given away. The stickers that the actor kept were put in white envelopes, while the stickers given to others were put in blue envelopes. The results indicated that both autistic and neurotypical children demonstrated altruistic tendencies (Peterson and Wellman, 2022). **(E)** The red figure represented the actor, the black figure at the table represented the experimenter, and the other black figure was the bystander of the same age as the actor. The experimenter pretended to spill water and required assistance. There were no bystanders in the left picture, two bystanders in the middle picture, and two bystanders in the right picture (but there were obstacles preventing them from helping the experimenter). The results showed that children were more likely to help the experimenter when there were no bystanders or when bystanders were unable to help (Plötner et al., 2015).

Human infants can also help even before the emergence of linguistic abilities. Warneken and Tomasello (2006) found that human infants would offer help in times of others’ needs, for example, when they saw that the experimenter had difficulty opening the door, they would help the experimenter open the cabinet door, which means that even toddlers (both prelinguistic and just-linguistic) have the tendency to help others solve problems. Sebastián-Enesco et al. (2013) believed that human pro-social behavior appeared in the early stage of development. In the study, 2.5-year-old toddlers could choose to pull the pro-social lever (both the actor and the recipient benefited) or pull the pro-self lever (only the actor benefited) (Figure 3A). The results showed that most of the subjects actively made pro-social choices, and after the role reversal, this pro-social behavior was not affected by the previous behavior of others, that is, whether the peer has made prosocial behavior before would not affect the expression of the toddler’s current prosocial behavior.

Can human toddlers sympathize and really care about the well-being of others? In order to verify this problem, Warneken and Tomasello (2008) observed the instrumental helping behavior of 20-month-old toddlers under different conditions (internal condition; praise condition; reward condition) by setting the situation where things fell to the ground and the experimenter could not reach them (Figure 3B), and they found that only verbal praise and no material reward stimulated more helping behavior of toddlers, and additional material reward would diminish the helping behavior of infants. At the same time, even if the cost of helping became significant (e.g., giving up the chance to play games), the probability of helping was not reduced. Warneken and Tomasello (2009) believed that instrumental help to others is human nature. They called the altruistic helping behavior of children as psychological altruism, that is, children transform other people’s goals into their own in order to benefit others (Warneken et al., 2007). Svetlova et al. (2010)’s research also supported this claim. In the helping scenario, toddlers (18 and 30 months old) needed to (temporarily) give up valuable items brought from home for helping others. For example, they needed to take their favorite hairpin to the experimenter to help the experimenter clip their hair, or to take their blanket to the experimenter who felt cold. The results showed that toddlers could not only understand the needs of the experimenter, they could even give up their favorite items to achieve altruism. Barragan and Meltzoff (2021) conducted a similar experiment on 19-month-old toddlers. The results showed that although toddlers had obvious possessive tendencies of their personal belongings, they would also give them to strangers who asked for help. In the study of helping behavior of 3-year-old toddlers, Engelmann et al. (2019) created a scenario in need of help. The subjects needed to choose whether to help their friends or non-friend peers, and can only chose to help one of them (Figure 3C). The results showed that toddlers would help their friends more, but would also help their peers who were not their friends. Peterson and Wellman (2022) studied 102 school-age children, and the experimenter gave them 10 pieces of stickers they liked most, which allowed them to freely control and keep or give to others. The ones kept by themselves were put in white envelopes, and the ones given to others were put in blue envelopes (Figure 3D). The results showed that both autistic and normal children had altruistic tendencies. In addition, in Hepach et al.’s (2012) study, it was found that children’s pupils dilated when they saw the experimenter in need of help. Once the experimenter was helped by themselves or others, the subjects’ pupil dilation weakened. The researchers believed that children’s pupil dilation reflected concerns about the misfortune of others, the motivation of helping behavior was intrinsic, and children sincerely cared about the well-being of others.

The above research shows that human toddlers can understand the needs of others and help out of caring for others. de Waal (2008) believed that empathy played an important role in the evolution of altruistic behavior. With the evolution of cognitive ability, the perception and matching of other people’s emotional state evolved into a more complex form, that is, caring about other people’s emotional state and trying to improve the other’s situation. This is also known as “sympathetic concern,” which is one of the forms of empathy. Toddlers between 1 and 2 years of age have this ability: they try to understand and care about the nature of other people’s pain, and to some extent make pro-social behaviors such as sharing, helping, and comforting (Zahn-Waxler et al., 1992).

### 3.4. Interim summary

In this chapter, we reviewed empirical evidence showing that rodents, non-human primates, and human toddlers may all have empathic concerns for the distress of the conspecific. They care about the conspecific, seek benefits for their conspecific, and help their conspecific in distress.

## 4. Why help others: Relieving personal distress

Personal distress refers to the propensity to experience pain upon exposure to the suffering or misfortune of others. It denotes an uncomfortable or uneasy emotional reaction to the grief or distress of others (Kim and Han, 2018). Sharing the fear or distress of the conspecific can cause personal distress. According to the model of negative emotion reduction, the individual’s helping behavior may also be to alleviate their own negative emotion (Cialdini et al., 1987), just as Darwin (1871) said in his famous “*The Descent of Man and Selection in Relation to Sex*” (p. 81): “*We are thus impelled to relieve the sufferings of another, in order that our own painful feelings may be at the same time relieved*”. However, Eisenberg (2010) believed that when individuals feel distressed, they are more inclined to escaping from the situation rather than choosing to help (also see Cameron et al., 2019).

In humans, the discussion of the relative importance of altruistic empathic concern versus egoistic alleviation of personal distress can be traced back to Batson et al. (1981) explored whether the motivation for helping behavior stems from the egoistic motivation of alleviating personal distress. In the experiment, a corn starch placebo and different instructions were used to manipulate the perceived emotional response (empathic concern or personal distress) of subjects when watching a confederate (named Elaine) being shocked based on the idea that watching others being shocked should elicit a reasonably high degree of both of these emotions and attributing the feeling of one emotion to the placebo would leave the other emotion to be perceived as the main one elicited by the situation. One group of subjects were told that the drug (placebo) would bring warm and sensitive feelings, making the group of subjects mainly perceive personal distress due to strong visual, auditory and other emotional contagion channels when watching Elaine being shocked (the empathic concern was wrongly attributed to the placebo); on the contrary, the other group of subjects were told that the drug (placebo) would bring uncomfortable feelings, which made the group of subjects mainly perceive empathic concern when watching Elaine being shocked (the personal distress was wrongly attributed to the drug). At the same time, the study also manipulated the difficulty of the escape condition. The goal was to investigate whether the subjects were willing to help Elaine and accept the remaining electric shock instead of her. The results showed that when the subjects perceived high empathic concern for Elaine, no matter how difficult it was to escape the situation, helping behavior was more likely to occur; however, when the subjects perceived high personal distress and the situation was easy to escape, the helping rate was significantly reduced. However, Cialdini et al. (1987) used similar paradigms and showed that enhanced personal distress was associated with empathy for the victim. Furthermore, they dissociated personal distress and empathic concern by leading the subjects to believe that their moods could not be altered due to the placebo drug, and found that in this case the subjects refrained from helping despite high levels of empathetic emotion. On the other hand, Stocks et al. (2009) believed that in Batson et al. (1981)’s study, the difficulty of escaping from the painful situation was manipulated by manipulating the number of times the subjects were required to watch Elaine being shocked, which provided physical escape but could not provide sufficient psychological escape. The author then manipulated the difficulty of psychological escape through memory preservation training or memory deletion training for the subjects. The experimental results show that empathy can evoke an altruistic motivation to alleviate the victim’s suffering rather than an egoistic motivation to alleviate their own aversion, even in the situation of easy psychological escape.

### 4.1. Evidences from studies on rodents

Among rodents, Lavery and Foley (1963) once tether-suspended a rat in the middle of the air to emit a painful scream in their study. They found that free rat would spontaneously learn to press the crossbar to help the suspended rat get rid of the pain, or press the crossbar to turn off the playback of the painful scream and end the existence of aversive sounds. The uncomfortable state of the suspended rat would make the free rat feel distressed, and when faced with events that cause aversion and their own distress, the rats would solve them by helping or avoiding (Knapska et al., 2010; Gonzalez-Liencres et al., 2014). In order to determine the motivation of the free rat’s helping behavior, Carvalheiro et al. (2019) improved the experimental device of Bartal et al. (2011) by adding a darkroom (Figure 1D) as an area for free rats to escape from the stressful situation. In the study, the subject’s cagemate was trapped in the restrainer of the light room. The subjects in the escapable group could escape to the dark room to relieve their distress, while the non-escapable group could not. They tested the latency of the free rat to open the door of the restrainer for the trapped cagemate, and found that compared with rats in the non-escapable group, rats in the escapable group took longer to help the trapped rats. By analyzing the free rat’s shuttle frequency between the dark and light areas, as well as comparing the stressful behavior (e.g., struggling) versus positive behavior (e.g., social investigation, proactive restrainer exploration) in both rats in the experiment, Carvalheiro et al. (2019) also found that the low anxiety level of the free rat and the positive behavior of both rats were conducive to the occurrence of helping behavior, as the higher the shuttle frequency of free rats between dark and light areas, the lower the anxiety of rats (Bourin and Hascoët, 2003). According to these data, the authors believed that the positive emotional states are conductive to the occurrence of helping behavior.

Knapska et al. (2006) described a rat model of emotional contagion (fear) in rats. Two rats were raised in pairs, one of which (the distressed rat) was either exposed to electric footshocks (the shocked group) or just exposed to a novel context (non-shocked group). Next, the distressed rat was sent back to the homecage to be reunited with its cagemate (the free rat). In the experiment, the approaching and exploratory behavior of the free rat and the performance of free rat in the acoustic start response (ASR) test were recorded and analyzed. The results showed that in the shocked group, the observer’s exploratory behavior toward the demonstrater was significantly enhanced, and in most cases the amygdala of the free rat was activated and the activation level was the same as that of the distressed rat, indicating that there was a strong emotional state transmission between the distressed rat and the observer, which caused the strong activation of the amygdala. In emotional contagion, sharing the fear of others may have the function of self-protection, using others as sentinels to improve preparation (Keysers and Gazzola, 2021). The anterior cingulate cortex and amygdala seem to play a necessary role in emotional contagion. This role seems to be conservative in rodents, macaques and humans, consistent with their evolutionary homology (van Heukelum et al., 2020; Paradiso et al., 2021).

The above research shows that the free rat can feel pain and fear of their peers in distress through emotional contagion, and such emotional contagion may have the function of self-protection. In addition, the free rat may initiate helping behavior to alleviate their own distress, but the positive emotional states of both rats are more conducive to the occurrence of help behavior.

### 4.2. Evidences from studies on non-human primates

McFarland and Majolo (2012) found that Barbary macaques tend to groom themselves more when they are exposed to a conspecific in a stressful state. In humans, scratching and self-grooming are self-directed behaviors that convey information related to individual psychological state (Ekman and Friesen, 1968). In order to understand the information function of self-directed behavior in non-human primates, Nakayama (2004) took Japanese monkeys (*Macaca fuscata*) as subjects to investigate whether watching the self-scratching behavior of the conspecific caused the negative emotional arousal of Japanese monkeys. The results showed that after watching the self-scratching behavior of the conspecific, they also began to self-scratch, indicating that self-scratching behavior has information function, it may involve the transmission of psychological states (or mere imitation). Therefore, the scratching and combing of non-human primates may also reflect the distress they feel through emotional contagion.

### 4.3. Evidences from studies on human early childhoods

A large number of studies on human infants and young children have shown that they may feel nervous, upset or sad because of the emotional response of individuals in pain, or even immerse themselves in sadness (Zahn-Waxler et al., 1992; Geangu et al., 2011; Roth-Hanania et al., 2011), indicating that infants and young children can feel the emotional state of others, and even cause personal distress due to emotional contagion. However, can personal distress drive infants to help? Zahn-Waxler et al. (1992) showed through a series of longitudinal studies that 2-year-old toddlers would have personal distress due to shared emotion when facing the pain of others, but this personal distress could not make them respond appropriately to the pain of others. For example, when they saw the mother’s leg injured, the baby would not comfort the mother, but stroked his own leg as a response. Through simulating an intense baby-crying scenario, Lin and Grisham (2017) investigated the preschooler’s response to baby crying. They found that although preschoolers had those micro-expressions indicating personal distress such as the downturned mouths, they by and large displayed infant-oriented empathic concern and helping behavior. Furthermore, the authors proposed that although personal distress could not directly predict helping behavior, it seemed to be related to infant-oriented process prior to help, such as inquiring about the reason why the baby cried, etc. In other words, the egoistic personal distress aroused by baby crying may provide the premise for preschoolers’ infant-oriented prosocial behavior. On the other hand, in the study of bystander intervention for preschoolers aged 3–5 years (Plötner et al., 2015), the researcher designed three conditions (Figure 3E). Condition 1 was that no bystanders available and children alone faced the experimenter in need of help; Condition 2 was that two bystanders were present; Condition 3 was that two bystanders were present, but obstacles prevented them from helping the experimenter, The results showed that children helped the experimenter more frequently in Condition 1 and Condition 3, which suggest that preschoolers would take into account their sense of responsibility when providing help. If they were the only person responsible for helping, but did not help, they might have guilt and personal distress. In addition, personality may play a vital role in helping behavior. MacGowan and Schmidt (2021) have studied the impact of children’s shyness on helping behavior, and found that shy children have less instrumental help. They concluded that shy children are more sensitive to social stimuli, and are more likely to focus on personal distress rather than on others, which makes children indifferent to others’ situation. Studies have also found that when infants and young children are in more intense fear states, their prosocial behavior become less (Spinrad and Stifter, 2006). Therefore, personal distress is a self-oriented emotional state that are aroused by others’ sufferings and may prepare infants and young children for further actions, but whether their actions are self-oriented or others-oriented may depend on their personality and maturity levels, such as self-other distinction (Steinbeis, 2016).

### 4.4. Interim summary

In this section, we reviewed emotional evidences showing that rodents, non-human primates, and human early childhoods can perceive others’ distress as their own through emotional contagion. For rodents and human early childhoods, moderate personal distress can help promote helping behavior.

## 5. Why help others: Pursuing social contact or other rewards

Rewards play a vital role in almost all aspects of life and constitute goal-oriented behavior (Rademacher et al., 2015). According to the “overjustification effect,” people typically engage in prosocial behaviors not because they expect some kind of direct benefit to offset their efforts but because they find it inherently rewarding (Chevallier et al., 2012). For example, the pursuit of social contact are intrinsic motivations, which may help the occurrence of behavior.

### 5.1. Evidences from studies on rodents

For a long time, there has been a debate about whether the motivation of rodent helping behavior is for social contact. Scholars have used different experimental paradigms for elucidating such possibilities. Bartal et al. (2011) once separated the free rat from the trapped rat by placing the door of the restrainer to an adjacent chamber (Figure 1E), so that the two could not make social contact after door opening. The results showed that even if they could not make social contact, the free rat would continue to help rescue the trapped rat, and they attributed the continuous helping behavior of the free rats to the empathic concern of the free rat, which were nevertheless modulated by the free rat’s social experience with the trapped rat (Bartal et al., 2014). Similarly, Cox and Reichel (2020) explored whether the free rat would help rescue the soaked rat when they could not socially interact. The results also showed that even without social contacts, the free rat would still help, but in this case the behavior was strongly modulated by efforts needed to help—if the efforts needed were high (e.g., the free rat needed to pull the chain 10 times to be able to rescue the soaked rat) then the free rat would stop to help. However, if they could socially interact afterward then high efforts would not hinder the helping behavior. Therefore, they concluded that the desire for social contact does play a role on top of empathic concern.

Some other scholars had more direct evidences in support of the role of social contact. Silberberg et al. (2014) repeated a critical part of Bartal et al.’s (2011) experiments in an ABA design. In Condition 1, after the free rat opened the door, the trapped rat would enter the adjacent chamber, and the two could not make social contact. In Condition 2, the released rat was in the same chamber as the free rat. Condition 3 repeated Condition 1. The results showed that when the free rat and the trapped rat could not make social interact (Condition 1), the latency to open the door (to help release the trapped rat) of the free rat became longer and longer as sessions went; when the two rats could then make social contact (Condition 2), the latency to open the door showed a downward trend; when the two rats were again unable to make social contact (Condition 3), unlike previous behavior of the free rat in Condition 1, the latency to open the door became shorter and the frequency of opening the door become higher. Furthermore, instead of immediately entering the adjacent compartment, the released rat spent more time in the restrainer, seemingly waiting for social interactions with the free rat. These behaviors seemed to indicate that the opening behavior of the free rat was not simply driven by empathic concern—it is the desire for social contact and social interaction with the trapped rat that becomes an important incentive factor for helping behavior to sustain (Lahvis, 2016; Hiura et al., 2018).

Similarly, Schwartz et al. (2017) used an E maze with a wet goal box and a dry goal box at its two ends to examine if the free rat’s helping behavior was due to the desire for social contact (Figure 1F). Both goal box had a trapped conspecific, but the wet goal box was filled with water (so that the trapped conspecific was also soaked). The results showed that the free rat preferred to approach the trapped rat in the wet goal box. In order to further explore whether the free rat preferred the wet rat or the water on the trapped rat, in the next experiment, the experimenters removed the trapped rat from both ends and found that the free rat still preferred the wet goal box. Their additional experimental results showed that the free rat preferred to approach the wet rat for two reasons: (1) for social contact with the released rat and (2) the reinforcing value of the pool of water. To further elucidate the role of social contact, Hachiga et al. (2018) used the E maze to compare the free rat’s preferences under three conditions. They found that when the two ends of the E maze were empty and trapped conspecific, respectively, the free rat preferred to enter the side with the trapped conspecific; when the two ends of the E maze were a trapped conspecific and another conspecific in a restrainer with the door open, respectively, the free rat had no preferences; when the two ends of the E maze were empty and a conspecific in a restrainer with the door open, the free rat preferred to enter the side with the conspecific (though not trapped). Based on these data, the authors believed that social contact is a powerful driving force for the free rat to open the restrainer. In one recent study, Heslin and Brown (2021) set three options, namely, the trapped rat, the free rat and an empty restrainer, for the free rat to choose. The results showed that the free rat preferred to approach the conspecific who was free to interact with, even if there were other conspecific who needed help at the same time. The experiment supported the important value for social contact. Ueno et al. (2019) obtained similar results in mice for the incentive value of the restraint tool. In addition, many studies have also confirmed the important incentive value of social contact/interaction (Calcagnetti and Schechter, 1992; Peartree et al., 2012; Kitchenham et al., 2019; Han et al., 2023).

In conclusion, the above studies show that rodents may be similar to humans. They have the need for social contact and prefer social contact. Social contact and interaction are their intrinsic motivation for maintaining helping behavior. Lack of social contact may hinder the occurrence of helping behavior.

### 5.2. Evidences from studies on non-human primates

Harlow and Zimmermann’s (1959) famous rhesus monkey experiment showed that physical contact played an even more important role in the development of baby monkeys than lactation. Only when there was a need for food, baby monkeys went to find their wire mothers, and most of the rest of the time they snuggled on their cloth mothers (Harlow and Zimmermann, 1959), which showed that primates like to contact with warm objects. At the same time, primates also tend to help for social contact. In Barbary macaques, after a fight, the defeated individuals tend to seek affiliative interactions by actively approaching bystanders to alleviate their post-fight anxiety. On the other hand, the bystanders also seek affiliative interactions by actively approaching the defeated individuals to exploit grooming opportunities, i.e., they seek the chance to comb their hair. All individuals in this context try to establish contact with social partners and benefit from the interactions (McFarland and Majolo, 2012). In addition, chimpanzees also prefer to cooperate. Greenberg et al. (2010) found that even if they themselves had received food or their peers had not shown any “request” for help, chimpanzees still tended to cooperate with their peers to pull the tether to obtain food. After a combat conflict, the phenomenon of “post-conflict affiliation initiated by a bystander,” also known as “reconciliation,” would appear. Researchers believe that the function of this phenomenon is to repair the relationship with valuable social partners (relationship repair), that is, after the combat conflict, the bystander would offer affiliation to either side (winner or loser) of the fight in representative of their friend in the fight. This can help the friend in fight reconcile with the opponent and maintain their interaction and cooperation (Wittig and Boesch, 2010). Indeed, social grooming and mutual grooming have been found to enhance the social connection of primates, and neuropeptides play an important role in this process of social connection (Dunbar, 2010).

In addition to social interaction, non-human primates can be motivated to exhibit helpful behavior by other rewards. Barnes et al. (2008) found that capuchin monkeys could also help human experimenters to fetch objects like chimpanzees do, but their helping behavior depends critically on their own costs and benefits. For example, they would only help if the helping was easy to do, and they would help much more if there was food reward in exchange.

In certain circumstances, obtaining a reward necessitates the joint efforts of multiple partners, such as cooperative hunting to subdue larger prey and increase the likelihood of a successful outcome. Research has shown that some non-human primates are capable of following norms of cooperation and reciprocity. Boesch (2012) observed the Taï Chimpanzee in the Taï Forest (Côte d’Ivoire, West Africa) and discovered that they exhibited the highest level of cooperation when hunting red adult colobus monkeys, displaying a complex and coordinated division of labor. In addition, the Taï Chimpanzee had meat-sharing rules, where spoils were often divided proportionally based on each individual’s specific participation in the hunt (Boesch, 2012). “Capturing the prey guarantees the highest reward, while ambushing and anticipating the prey movements, which often are necessary contributions, are highly rewarded as well. Performing other, less decisive roles does not increase meat access.” These rules are socially enforced, indicating that the Taï Chimpanzee has developed specific cooperation norms to ensure that everyone is appropriately rewarded for their contribution to the hunt. Furthermore, a study explored cooperative reciprocity in capuchin monkeys. Two monkeys were separated by a mesh partition, with a bowl (either empty or with apple slices) in front of each monkey, each bowl accessible to one monkey by pulling the tray toward itself using one of two protruding bars. In the cooperative effort tests, both monkeys had to pull, but only one bowl was baited. In the solo effort control tests, only one monkey had access to a pull bar and a baited bowl. The amount of apple slices shared was measured. The results revealed that significantly more food pieces were shared in the cooperation tests compared to the solo effort tests (de Waal and Berger, 2000). This phenomenon has also been observed in other studies where capuchin monkeys actively distributed better food to those who helped them access rewards in joint tasks (Takimoto and Fujita, 2011). These studies suggest that capuchin monkeys are sensitive to the efforts of others, may have certain reciprocity rules, and voluntarily share treats with other monkeys who helped to obtain them (de Waal and Berger, 2000; Takimoto and Fujita, 2011). Adherence to reciprocity rules can increase cooperation’s sustainability.

In short, in the study of primates, we can see the important role of social contact. Additionally, it has been found that receiving other types of rewards can also serve as a trigger for helping behavior. Non-human primates have been observed to cooperate when the achievement of a reward requires joint effort, and they adhere to specific rules of cooperation by sharing rewards with those who contributed to the cooperative task.

### 5.3. Evidences from studies on human early childhoods

In human infants and young children, the impact of physical contact should not be underestimated. Evidence shows that skin contact and breastfeeding at birth optimize psychophysiological functions (Saxton et al., 2014). By studying the interaction between 146 premature infants at the age of 3 months and their parents, it was found that the parents who received skin-to-skin contact were more sensitive to the baby and had less negative impact on the baby, the baby had more frequent intimate contact with parents, the feelings between spouses were closer, and the family style was more cohesive (Feldman et al., 2003). In addition, some scholars believe that early mother-infant contact can help babies construct a prosocial schema (Su and Su, 2018). In other words, physical contact between mothers and infants promotes the pursuit of social connection and prosocial behavior. For crying babies, the mother’s embrace can make the baby stable quickly. Early skin contact with the mother or caregiver may be the origin of the desire for social contact.

Physical contact has an indispensable impact on infants and young children. However, will infants and young children help in order to make social contact with others? Pletti et al. (2017) questioned children’s motivation to really care about the well-being of others, and believed that children’s helping behavior may also be for social contact. Svetlova et al. (2010) believed that infants and young children showed instrumental help behavior, partly because they liked to participate in cooperative actions and felt happy in cooperation. A study by Cortes Barragan and Dweck (2014) confirmed this hypothesis. They used a “reciprocal game” or “parallel game” to observe the effect of the game on the helping behavior of 1- and 2-year-old infant subjects. The results showed that infants were more likely to help the experimenter after playing with each other than playing parallelly. The study by Ulber and Tomasello (2020) also found that compared with the situation when they needed to help alone (assisting another person open a locked box), when they needed to cooperate to complete the task of helping (cleaning the room together), 2- or 3- year-old children’s helping behavior were much more frequent. In addition, the study points out that children showed a reciprocal preference. When the experimenter clearly expressed that they would help the children, children’s helping behavior toward the experimenter significantly increased. What’s more, the frequency of physical contact of children of different ages is different. Compared with older children, younger children have more physical contact, and the results of physical contact are more positive (Cowen et al., 1982, 1983). In addition, studies found that infants and children would help in order to obtain rewards. For example, Kenward et al. (2015) found that 4-year-old children tend to help those who can give back to themselves—if others cannot give back, children are unlikely to help. Hepach et al. (2023) found that 2-year-old children would provide help mainly out of concern for the well-being of others, while 5-year-old children have developed additional strategic motivation for helping others, and they would help mainly to improve their reputation. When 5-year-old children’s behavior were observed by adults (so that their reputation may be threatened), helping led to their more positive emotions.

Research on children has demonstrated that social interaction, in addition to physical contact and other rewards, plays a significant role in motivating their helping behavior. Reciprocity, a powerful social norm that stabilizes social relationships, also regulates children’s helping behavior (Wörle and Paulus, 2019; Myslinska Szarek and Tanas, 2022). However, understanding reciprocity requires a certain level of cognitive ability. Studies have shown that young children as young as 3 years old can display prosociality toward those who have benefited them (Vaish et al., 2018). Still, it is not until they gain more experience or develop other cognitive skills that they become regulated by reciprocity. For example, Warneken and Tomasello (2013) investigated whether young children would adjust their helping or sharing behaviors based on their peers’ actions in a game scenario by role reversal. The results showed that 3.5-year-olds were more likely to share if their peers had previously shared with them, but the partner’s previous help did not affect their helping behavior. Similarly, Myslinska Szarek and Tanas (2022) found that preschoolers aged 4–6 years could understand and follow reciprocity norms, using it as a motivation for helping behavior. In their study, preschoolers were presented with a scenario where a protagonist had previously helped non-friends and needed help themselves. The results showed that 60% of the participants chose to turn to the non-friends for help, indicating that they expected reciprocal behavior from those who had benefited from their prosocial behavior. Similarly, Wörle and Paulus (2019) found that 5–6-year-olds can endorse and follow reciprocity norms.

Overall, research on human early childhood suggests that social interaction and reward acquisition play a significant role in motivating their helping behavior. As their understanding develops, they can follow reciprocity norms, helping those who have helped them and expecting reciprocity from those they have helped.

### 5.4. Interim summary

In this section, we reviewed empirical evidence showing that rodents, non-human primates, human infants and young children have a desire for social interaction, and social contact or the expectation of rewards alike can help promote the occurrence of helping behavior. Simultaneously, certain non-human primates and young human children have demonstrated the ability to abide by norms of cooperation and reciprocity in regards to social interaction and reward acquisition.

## 6. Summary and discussion: A circle-layered model for the motivation to help

**FIGURE 4:**
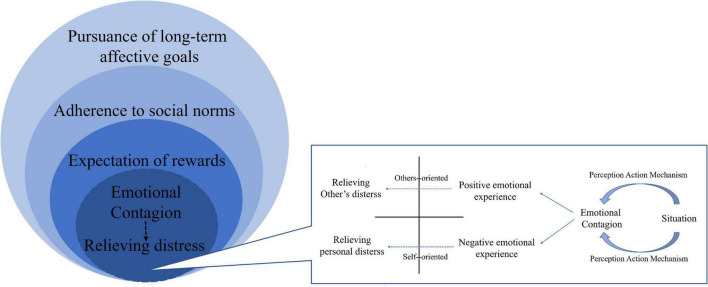
The circle-layered model of motivation for helping behavior. This figure illustrates the circle-layered model of motivation for helping behavior. According to this model, at the core of helping behavior is the emotional contagion mediated by the perception-action mechanism, which allows individuals to perceive and understand the emotional state of the individual in need of help. This mechanism leads to two different emotional experiences, empathic attention to others or personal distress in oneself, which can motivate individuals to help alleviate their own distress or that of others. The second-layer motivation for helping behavior is the expectation of rewards, which includes both extrinsic and intrinsic rewards. Extrinsic rewards can include social interaction, material rewards, or praises, while intrinsic rewards can include the positive experiences and sense of pleasure generated by helping others. These rewards not only act as positive outcomes of helping behavior but can also be transformed into a motivation for helping behavior. The third and fourth layers of motivation for helping behavior are mediated by the more advanced affective-cognitive system. These layers include abiding by social norms and pursuing long-term affective goals, such as pride and a sense of meaning. Overall, this model suggests that the motivation of helping behavior may evolve and develop from the emotional-behavioral system to the affective-cognitive system, and from the inner layer to the outer layer, which is likely shared by humans and non-human mammals.

In the preceding discussion, we have reviewed three primary motivations for helping behavior that are currently a topic of much debate in the literature. Our analysis has revealed that the motivation for helping behavior is deeply rooted in both ontogenetic and phylogenetic development, with empathic concern, the desire to alleviate personal distress, and the desire to make social contact with others being common in rodents, non-human primates, and human early childhoods. Building on this and previous research, we propose a circle-layered model for the occurrence and development of motivation for helping behavior, as illustrated in Figure 4. At the core of this model is a simple perception-action mechanism (PAM), with the inner layers comprising motivations for helping behavior that are proximal and dominated by the emotional-behavioral system, and the outer layers comprising motivations that involve ultimate causes and are dominated by the affective-cognitive system. We believe that our model can enhance the understanding of helping behavior, inspire further research in this field, and potentially predict the presence of helping behavioral motivation in humans at various developmental stages.

The development of behavior is characterized by a distinction between proximal and ultimate causes, as posited by Mayr (1961). The proximal cause refers to the biological tendency and psychological mechanism underlying behavior, elucidating how it functions, while the ultimate cause emphasizes adaptation and coordination between individuals and their environment, explaining how behavior is determined by specific genetic information from evolutionary selection. According to Preston (2013), the ultimate cause of helping and altruistic behavior originates from the parental rearing behavior involved in offspring care. Mammals must respond promptly to the emotional signals and needs of their offspring, and this practice forms the foundation of many forms of helping behavior that extend beyond future generations to strangers. Furthermore, when mammals can differentiate between their own and others’ states (Hoffman, 1998), they can resonate with the distress and happiness of their conspecifics through primitive emotional contagion (Panksepp, 2011). This emotional response core is the “Perception-Action Mechanism” (PAM), which has been preserved in evolutionary development (de Waal and Preston, 2017).

Proximally, in situations requiring help, individuals process information in the environment spontaneously, decode the conspecific’s emotions, achieve state matching, and generate emotional concern, as proposed by de Waal and Preston’s (2017) PAM model of empathy, which also underlies the premise for helping behavior. However, the ability to perceive others’ states through emotional contagion does not necessarily lead to helping behavior, which also requires motivation. Emotional empathy may not only lead to the motivation of helping behavior centered on others and paying sincere attention to the well-being of others, but also may lead to the motivation of helping behavior in order to alleviate their own distress due to excessive emotional arousal. In addition, the expected reward is also a motivation for helping behavior. Social interaction and contact have incentive value, accompanied by the transmission of oxytocin, dopamine, and other neuropeptide signals, and the activation of brain areas such as the ventral striatum and ventromedial prefrontal cortex (Marsh et al., 2014), which bring about a positive emotional experience. These proximal motivations of helping others in the context of helping behavior involve empathic concern, motivation to alleviate one’s own distress, and motivation to attain rewarding social contact, which are shared by humans and many non-human mammals. This is also consistent with Lahvis’s (2016) view on biological altruism and help, which emphasizes social motivation and empathy as the proximate causes of altruism and help. Social motivation and contact can bring positive feelings to individuals and make them more susceptible to the influence of others’ behavior. Empathy, on the other hand, enables individuals to experience emotional states that are suitable for others, and it also has predictive value in terms of sensing potential dangers from social cues. Therefore, altruism is influenced by both social motivation and empathy. Lahvis coined the term “the Camaraderie Effect” to describe the positive influence that helping behavior can have on both the helper and the recipient of help.

As the affective-cognitive system develops to a higher level, individuals may also be motivated to comply with social norms (Siposova et al., 2021) and to gain and maintain social identity (Oarga et al., 2015). Besides, they may consolidate their past interactive experiences with the environment and form a unique personal belief system where the motivation for helping others comes from the long-term satisfaction of internal needs. Over time, these affective goals, such as finding meaning in life, may further evolve as the ultimate mechanism for helping behavior. In general, an individual’s motivation for helping behavior may follow an evolutionary and developmental sequence, starting from the emotional-behavioral system and progressing toward the affective-cognitive system. This sequence constitutes the circle-layered model of motivation for helping behavior, which we will now describe in more detail, starting from the inner layer and moving outwards.

### 6.1. The basic core of helping: Emotional contagion mediated by the perception-action mechanism

The basic process of helping behavior is consistent with the PAM model for empathy. According to PAM, when an individual pays attention to others, he or she obtains information about others’ state, feelings and other information, which will automatically activate the specific representations related to the observer. The nervous system maps this information to the individual representations, and generates “self-other overlap” to sense and understand the feelings of the other party, so as to match the emotional state (de Waal, 2008, 2012; Preston and Hofelich, 2012; de Waal and Preston, 2017). Neural “representation” refers to the distributed neural codes in the brain, which are often formed through experience, and can also organize discrete experiences into abstract knowledge, concepts, memories, connections, etc. When there is no highly similar experience, the representation plays its role through bottom-up perception and high-level affect. At this time, the “self-other overlap” is an automatic process that occurs unconsciously and can only be understood abstractly. However, if the observer has experienced similar states and experiences, the “self-other overlap” is the process generated by memory extraction or subjective experience at the neural level (Preston and Hofelich, 2012). Therefore, the greater the similarity and familiarity with the other party, the more consistent the representation, the more accurate the perception and understanding of the other’s emotional states.

In short, PAM describes the process of spontaneous information processing and decoding of other’s emotions, generating emotional contagion, and understanding of other’s states. This is the basic process of helping behavior, and past experiences and personal distress level may affect this process.

### 6.2. Motivation of helping behavior dominated by the emotional-behavioral system

#### 6.2.1. Alleviation of other’s distress and their own distress—The first layer

In the helping situation, the emotional contagion mediated by PAM forms the perception and understanding of the emotional states of the individual in need of help, which is the basis for helping behavior to occur. It usually brings two different types of emotional experience to the observer, that is, the empathetic attention directed to others and the personal distress directed to self (Batson, 2009; Singer and Klimecki, 2014; Graziano and Habashi, 2015; Yue et al., 2018). These two types of emotional experiences will generate different motivation for helping behavior.

Empathic attention is a positive emotional experience as it focuses on others and is based on the general and calm perception and understanding of the emotional states of others. It expresses the real concern for the well-being of others. Under this kind of emotion, individuals have the motivation of helping others to alleviate their distress, which increases the possibility of helping or altruistic behavior (FeldmanHall et al., 2015; Winczewski et al., 2016). Personal distress is a negative egoistic emotional experience, based on the perception and understanding of the emotional states of others, and combined with their own past experience, resulting in excessive emotional arousal. Under this condition, the degree of self-other overlap is higher, and individuals have the motivation to alleviate their own distress, and relieve their own tension and pain by helping to end the distress of others. Of course, the way to alleviate their own distress is not always to help. If the distress is possible to escape, the individual may also choose to escape the distress rather than help.

No matter what kind of motivation for helping behavior, it shows that individuals must share the pain of others on the basis of PAM-mediated emotional contagion, that is, feeling distress is a necessary condition for implementing helping behavior. The study of Bartal et al. (2016) proved that the free rat needs a negative emotional state to open the restrainer to rescue the trapped rat. In their study, the application of anxiolytic drugs to free rats will significantly reduce the frequency of opening the door for the trapped rat. Similarly, Carvalheiro et al. (2019) found that the free rat’s helping behavior was impaired when providing the opportunity to escape by entering an adjacent dark chamber. In any scenario, the distress one feels may be directly from the distress of others, or it may be from their own. It should be noted that excessive sharing of others’ distress through emotional contagion may cause individuals to fall into the state of freezing, thus preventing them from taking action and hindering helping behavior. Therefore, only the activation of a moderate distress state can help promote helping behavior. In support of this notion, Bartal et al. (2016) found that too low or too high corticosterone levels are not conducive to the successful helping of rats. The effect of distress on prosocial behavior follows an inverted U-shaped curve, and moderate distress is most helpful (Buchanan and Preston, 2014; Muroy et al., 2016).

#### 6.2.2. Expectation of rewards—The second layer

Rewards play an important role in all aspects of life. They are the basis of goal-oriented behavior and motivation (Rademacher et al., 2015). Rewards brought by helping behavior can directly or indirectly benefit the helper and reinforce helping behavior. Therefore, expectation of rewards constitute the second-layer of the motivation for helping behavior, which can include the intrinsically experienced pleasure generated by oneself, opportunities for social contact/interaction with others, expected material returns, praises and other extrinsic rewards. They are not only the positive results of helping behavior, but also the motivation or expectation for future helping behavior to occur in similar circumstances after establishing the “helping-rewards” relationship. Although the manifestation of motivation at this level are different, they all have positive rewarding attributes and have similar neurophysiological basis. For instance, within the central nervous system, the manifestation of this emotional response is accompanied by the transmission of neuropeptide signals, including oxytocin and dopamine, the activation of brain regions related to reward, such as the ventral striatum and ventromedial prefrontal cortex, and the regulation of the amygdala for negative emotions (Marsh et al., 2014). It is important that during the expectation of rewards period, the incentive value of rewards depends on cue salience to produce the approaching behavior toward the ideal goal (Berridge and Robinson, 1998), which also follows the perception-action mechanism.

Pro-social behavior and helping behavior promote social connection and social integration (Aknin and Human, 2015; Aknin et al., 2018), and contribute to the establishment and maintenance of social relations (Martela and Ryan, 2016). In the process of helping behavior, one can socially interact with the help recipient and get social contact opportunities, which is a powerful driving force for helping behavior. On one hand, social contact and physical touch make the helper feel good and have intrinsic reward value. Research shows that there are type-C tactile afferent fibers on human hairy skin, which make people experience pleasure in gentle touch and intimate social behavior (Löken et al., 2009). This is a reward from the peripheral nervous system, which enhances people’s desire to act in this way in the future (Löken et al., 2009). Similarly, the reward of the central nervous system can also be obtained in social interaction. Research shows that rodents can promote the expression of oxytocin in oxytocin neurons in the paraventricular hypothalamus (PVH) through the contact of body and whisker during social interaction, and induce the increase of oxytocin firing, which dipeptidic circuit mediates the social behavior of physical pleasure (Zheng et al., 2014; Yu et al., 2022). On the other hand, according to the basic-psychological-needs theory of motivation, relationship (feeling that you need to be connected with others) is one of the three basic psychological needs, reflecting the expectation of connecting with others, conducting social interaction, feeling cared for, relied on, and feeling close relationship with others (Deci and Ryan, 2000). The satisfaction of this need can promote mental health and happiness. After experiencing the benefits of social interaction, social interaction motivation will be further expanded in the process of socialization and transformed into deeper motivation, such as establishing social connections, improving relationships, expanding social networks, etc. In order to benefit from new relationships, organisms will gradually evolve, even with larger groups, or with completely unfamiliar conspecifics (Allen et al., 2022). In fact, the pursuit of social connection and social integration is equivalent to the need for a sense of belonging. Social animals are generally driven by a sense of belonging at the root, and have a strong desire to form and maintain a lasting interpersonal attachment. They seek frequent and emotional positive interaction in long-term loving relationships (Baumeister and Leary, 1995).

Although helping behavior is to solve problems for others and benefit others, helping behavior may also be accompanied by rewards from others. Anticipated behavior may be rewarded extrinsically, and may also become the motivation of helping behavior. However, providing extrinsic incentives may be counterproductive to helping behavior. Autonomous motivation rather than controlled motivation is more conducive to the cultivation of helping behavior (Warneken and Tomasello, 2008). According to Deci and Ryan (2000)’s theory, the self-determined behavior is a relatively independent, freely actionable and fully recognized behavior of an individual, rather than a behavior coerced by external forces or expectations. It can be seen from this that the expected extrinsic reward is not conducive to maintaining helping behavior. Only the behavior driven by curiosity, interest and other intrinsic motivations can promote happiness and sustain helping behavior.

In addition, the rewarding effect of personal distress relief is different from that of seeing others’ distress relief. The relief of others’ distress is a substitutable reward, while the relief of their own distress is a direct personal reward. Therefore, the rewarding effect of the former may be weaker than the latter. Morelli et al. (2015) found that compared with vicarious rewards, personal rewards preferentially activate nucleus accumbens (NAcc) because they are the result of the individual’s direct feelings after action, while the vicarious reward tasks usually involve passive reward reception after observation. In addition, although the ventromedial prefrontal cortex (vmPFC) usually responds to both vicarious and personal rewards, when dealing with vicarious rewards, vmPFC would uniquely integrate regional information that participates in social cognition, and more activated regions related to value calculation and psychologization. Therefore, observers who see the distress of the other party relieved may calculate the value of these results, just as they have obtained personally. However, they may not experience the excitement as receiving their personal rewards, thus reducing the activities in NAcc.

### 6.3. Motivation of helping behavior dominated by the affective-cognitive system

#### 6.3.1. Adherence to social norms—The third layer

Social norms refer to the rules of conduct that the social members of the same group jointly observe and implement (Hechter and Opp, 2001). These rules are formed based on widely accepted beliefs, and the process of their formation is not yet fully understood. However, the culture-gene coevolutionary models of social behavior may help explain how the motivation to comply with social norms arises (Chudek and Henrich, 2011). After human ancestors discovered the benefits of cooperation, helping and prosocial behavior, this adaptive information began to accumulate gradually in generations, and finally encoded a more complex and more survivable phenotypic repertoire. They encoded in group memory, became group experience, became part of genes, and evolved into norm psychology.

Social norms can be divided into two categories: descriptive social norms and injunctive social norms. Descriptive social norms refer to how most individuals behave in a specific situation, while injunctive social norms refer to certain behaviors that are either clearly accepted or prohibited by society (Cialdini et al., 1990). These two types of norms also influence helping behavior. Descriptive norms affect helping behavior by conveying appropriate and effective behavioral information and by promoting internalization and conformity to common norms. Vicarious learning through feedback from others’ behavior also promotes observational learning and encourages helping behavior. For instance, studies have shown that tufted capuchin monkeys (Cebus apella) can socially evaluate humans based on witnessing third-party interactions involving helpful interventions or failure to help (Anderson et al., 2013a,b). Infants also show sensitivity to third-party social interactions, reflecting the early developmental characteristics of human social cognitive structure and its fundamental role in early social and moral development (Farris et al., 2022). Injunctive norms influence helping behavior by clearly stipulating social recognition and expectations of behavior through social rewards or punishment (Lay et al., 2020). While people may donate or help because of pressure to conform to social norms even when unwilling to (Dunning et al., 2016), motivation to comply with social norms is activated when people recognize the common expectations of social groups. Importantly, they have learned that violating social norms can lead to expectations of social exclusion and punishment.

Compliance with social norms in helping behavior requires cognitive recognition of social behavior standards and a sense of obligation to help. Among young children, public knowledge can increase the sense of obligation to help those in need, particularly when there is common knowledge between the helper and the recipient (Siposova et al., 2021). Abiding by social norms also has emotional components, including the sense of pride of being obeyed, and the sense of shame and guilt of not obeying (Schwartz and Howard, 1982). In countries where helping behavior is a strong social norm, helping behavior is closely related to life satisfaction and makes people feel good about their behavior (Oarga et al., 2015). Basile et al. (2011) demonstrated that the neural activity involved in guilt generation corresponds to the neural network associated with opinion selection, suggesting that the primary function of guilt is to facilitate the selection of opinions. Guilt and shame are emotions that pertain to the advancement of social and others’ interests, and they represent negative assessments of one’s own breach of social norms. Observing social norms is an important facet of social communication and interaction, reflecting the quality of individuals. With regards to helping behavior, individuals often abide by general social responsibility norms of fairness and justice to assist those in need. Furthermore, individuals adhere to the norms of social reciprocity to maintain equilibrium in relationships and help those who have aided them, as evidenced by observations of Taï Chimpanzees and capuchin monkeys (Boesch, 2012; Anderson et al., 2013a,b). In addition, closely tied to the observance of social norms is the observance of moral norms. Human beings possess moral beliefs, virtues, and values. When moral virtues are integral to an individual’s identity, there is motivation to act in accordance with a moral sense (Hardy, 2006). Thus, when virtues such as “helping others” and “integrity and kindness” form the core of one’s identity, a stronger sense of responsibility ensues, and a desire to remain consistent with these virtues and act according to moral norms emerges. Whether adhering to social or moral norms, they promote the internalization of values and the sustained occurrence of helping behavior.

Research has demonstrated that the motivation to comply with social norms can be acquired during the process of socialization, with children developing a preference for social norms as early as 7–8 years old (Fehr et al., 2008). Different brain regions are implicated in implementing and dealing with violations of social norms, with the right anterior cingulate gyrus and medial frontal gyrus (BA10) being crucial for implementation and the insula, dorsolateral and dorsal cingulate cortex playing a key role in dealing with norm violations (Zinchenko and Arsalidou, 2018). Two brain systems have been identified as involved in normative decision-making regarding fairness: the affective/intuitive system (System 1) and the computational/deliberative system (System 2) (Harlé and Sanfey, 2012; Feng et al., 2015). The affective/intuitive system comprises the anterior insula and ventromedial prefrontal cortex and is responsible for punishing those who violate norms, while the computational/deliberative system includes ventrolateral prefrontal cortex (vlPFC), dorsomedial prefrontal cortex (dmPFC), left dorsolateral prefrontal cortex (dlPFC), and rostral anterior cingulate cortex (rACC), and is responsible for cognitive control, inhibition of personal interests, and conflict resolution.

It is worth noting that empathy and compliance with social norms can interact and jointly affect helping behavior. Research has indicated that under high empathy, the influence of norms on helping behavior is diminished (Lay et al., 2020). In contrast, when empathy levels are low, social norms exert a greater influence on guiding helping behavior. Additionally, individuals with high empathy are more inclined to help and activate the prefrontal and parietal regions more during the helping process (Hu et al., 2015).

#### 6.3.2. Pursuance for long-term affective goals—The fourth layer

The helping behavior induced by the motivation to comply with social norms may be stable, but it is important to acknowledge that this behavior may not necessarily stem from the personal will of the helper or be the individual’s own choice. As individuals further develop their affective-cognitive system, they may form a unique personal belief system where the motivation for helping others comes from the satisfaction of internal needs. Such individuals may pursue long-term affective goals and attach importance to the value and significance of helping behavior. While the helping behavior under this motivation is an independent choice, it is crucial to note that the helping behavior is also more stable because it leads to lasting satisfaction.

Survival is not logical. Species evolved with different affective profiles for different survival needs (McShea, 2017). Different species have different survival goals because they have different affective profiles. Therefore, the formation and development of the motivation to pursue long-term affective goals may also have evolutionarily ultimate and proximal causes. At the ultimate end, when resources were scarce, helping behavior was often unwise as the helper needed to pay a certain cost that could even cause severe danger to the survivor. However, helping behavior could also bring internal benefits to the helper, such as a sense of warmth, pride, and the significance of life. These emotional benefits may lead to a positive cycle of physical and mental interaction through the self-motivational effect (Xie et al., 2017). When the internal utility gain brought by helping behavior to helpers becomes greater than the external utility cost consumed by helping behavior, helpers may have more advantages than self-interests, making them more likely to win in natural selection and promote the preservation of helping behavior in groups. Similarly, among groups, helping behavior may increase the adaptability of groups and help people evolve among groups. At the proximal end, the affect felt in the helping behavior may influence future helping decision. Such affect can promote cognitive reflection, promote learning, and guide future behavior by providing feedback and stimulating retrospective appraisal of actions (Baumeister et al., 2007). When people learn to predict the affective feedback of helping behavior, they may change their behavior to seek the feedback they like, or to avoid the expected negative outcomes. Driven by the motivation to pursue long-term affective goals, helping behavior may more often bring positive affect and positive self-cognition, including warm perception of the surrounding environment.

To be more specific, helping and altruism can activate people’s psychological process, leading to people’s warm perception of the surrounding environment. It is important to note that people who help feel warmer about their surroundings than those who refuse to help or have no chance to help (Hu et al., 2016). In the process of helping or after helping, the expected results are accompanied by positive affect, such as joy, grace, serenity, interest, hope, pride, emotion, inspiration, awe, and love (Fredrickson, 2013). Pride, for instance, can promote the pursuit of valuable social behaviors and is universal in human nature (Sznycer et al., 2017; Tracy et al., 2020). It is an affect with self-consciousness and moral emotion. It only appears when infants and young children develop self-consciousness and develop the ability to evaluate themselves according to external standards (Lewis, 2003). Pride is different from happiness, which is related to immediate enjoyment (Eyal and Fishbach, 2010), while pride (and guilt) can regulate the pursuit of long-term goals, which is related to the realization of long-term goals. Therefore, the pursuit of positive affect, especially one that regards helping others as a sense of pride, may help shape helping behavior.

Besides bringing positive affect, helping behavior can also bring higher affective value and a sense of meaning. Helpers can feel the “warm glow” in their helping behavior (Andreoni, 1989), sensing that they are a good person or are happy to see others being helped, just like “Doing Good, Feeling Good”. Aknin et al. (2012) found that children under the age of two can experience greater happiness by giving food to others than by themselves, even if it had costs, demonstrating that the sense of help—”warm glow”—can be observed in early development. Moreover, Martela and Ryan (2016) also showed that paying close attention to the welfare of others would enhance happiness. In addition to the pursuit of positive emotions, helping behavior brings several benefits to helpers, including happiness in giving, enhanced self-esteem, improved self-efficacy, and promoting mental and physical health, as well as interpersonal harmony (Crocker et al., 2017).

In conclusion, the pursuit of positive affect such as pride, a sense of meaning, and happiness in life can promote individuals’ long-term goal and value orientations. When individuals are motivated to help others based on such long-term affective goals, they may be more inclined to prioritize the benefits of others, and approach tasks with the intention of achieving these goals. Consequently, this motivation can increase the likelihood of individuals being willing to assist others, thus fostering prosocial behavior. Nevertheless, this layer is still speculative and calls for further research in this area.

### 6.4. Learning, habituation, and persistence of helping behavior

In the preceding paragraphs, we presented the circle-layered model of motivation for helping behavior and highlighted how the distinct motivational layers can facilitate the manifestation of helping behavior. Moving forward, we aim to explicate how helping behavior is influenced by diverse motivational factors, including the learning mechanism and internalized persistence mechanism of helping behavior.

As mentioned previously, the core of helping behavior is emotional contagion, which is mediated by the PAM and emphasizes the impact of environmental stimuli on an individual’s emotional state. Emotional distress of others is transferred to the observer, which promotes a similar emotional state. Once the observer performs an accidental response to help the other party, the resulting experience affects the reinforcement or extinction of the behavior. While individuals may not initially have a strong sense of motivation when helping others, the implementation of helping behavior can lead to positive outcomes such as satisfaction, enjoyment, self-determination, behavioral consistency with cultural norms or belief systems (Kwasnicka et al., 2016), all of which contribute to the development of incentive factors for future actions. These incentives include motivation to alleviate personal or others’ distress, motivation to obtain rewards, motivation to comply with social norms, and motivation to pursue long-term affective goals. This transformation process from experience to motivation also leads to the gradual formation of an individual’s abstract social cognition. From this perspective, helping behavior can be viewed as a form of “learned altruism,” as it strengthens helping behavior and enables individuals to learn to help by connecting with positive results, which is a common learning mechanism among both human and non-human mammals. Furthermore, helping behavior can also be acquired through social learning or cultural transmission, where parents guide and correct behavior to help children acquire moral rules and attitudes (Ashcroft, 2001; Lehmann et al., 2008), a learning mechanism found in more advanced animals. The theory of habitual learning suggests that the formation of habits relies on repetition and reinforcement. Through repeated positive reinforcement, helping behavior can become habitual and internalized within individuals’ behavioral schema. Once the behavior becomes a habit, contextual cues can automatically trigger helping behavior, bypassing conscious decision-making processes. Furthermore, both humans and some higher non-human primates display a consistent motivation to help others in order to conform to social norms or to avoid punishment. As the affective-cognitive system develops further, higher-level motivations for helping behavior, such as pursuing long-term affective goals, may emerge. These motivations can be integrated into the moral system, becoming internalized as moral virtues such as “kindness and integrity” (Curry et al., 2018). When helping behavior is consistent with an individual’s internal beliefs and values, it can foster the persistent and stable occurrence of such behavior. As a result, helping behavior that becomes a habit, virtue, belief, and value can develop into a powerful and long-lasting force.

## 7. Conclusion and future direction

In the preceding chapters, we have primarily focused on helping behavior and its underlying motivation. We have reviewed various potential motivations for helping behavior from a comparative perspective, described how different motivations facilitate the occurrence of helping behavior, and proposed a circle-layered model for the motivation to help, which evolves from the emotional-behavioral system to the affective-cognitive system, and develops from the inner layer to the outer layer. This model has recently received support from studies such as Eisenberg et al. (2016), who put forward a theoretical framework of motivation for prosocial behavior. They proposed that empathy and adherence to personal norms such as fairness and equality are altruistic motivations, while raising self-esteem, reducing aversion, receiving praise and rewards, and avoiding punishment are egoistic motivations. Building on this model, Hao and Du (2021) conducted an experiment investigating the instrumental helping behavior of preschool children through tasks that involved arranging cards for sick peers. Their study demonstrated that empathy, compliance with moral rules, receiving praise, and rewards can all promote preschool children’s helping behavior, indicating the diversity of their motivations. They also investigated how motivation develops with age and found that altruistic motivation develops faster than egoistic motivation, and emotional altruistic motivation develops faster than cognitive altruistic motivation. Most 5-year-old children help others out of empathetic motivation, while only a few 5-year-old children help others out of motivation to comply with moral norms. This study supports the idea that the motivation to help others may develop from the emotional-behavioral system to the affective-cognitive system, from the inner layer to the outer layer, as proposed in this paper.

However, more experimental evidence is necessary to validate the model and explain the motivation for helping behavior. It is also important to note that our discussion has primarily focused on the motivation for helping behavior and has not included other complex psychological processes involved in helping, such as attention, cost appraisal, decision-making, and outcome expectation (Nartova-Bochaver, 1991; Decety et al., 2016). Therefore, future research should aim to provide a more complete picture of the neural and psychological processes pertaining to the occurrence and maintenance of helping behavior.

## Author contributions

BY initiated and supervised the project. Y-QC and SH wrote the initial draft of the manuscript. All authors critically revised the manuscript for submission and approved the final version of the manuscript.
